# The Link Between Periodontitis and Rheumatoid Arthritis: A Periodontist’s Perspective

**DOI:** 10.1007/s40496-014-0040-9

**Published:** 2014-12-24

**Authors:** Jeffrey B. Payne, Lorne M. Golub, Geoffrey M. Thiele, Ted R. Mikuls

**Affiliations:** 1 Department of Surgical Specialties, Division of Periodontics, College of Dentistry, University of Nebraska Medical Center, Lincoln, NE USA; 2 Department of Internal Medicine, College of Medicine, University of Nebraska Medical Center, Omaha, NE USA; 3 Department of Oral Biology and Pathology, School of Dental Medicine, Stony Brook University, Stony Brook, New York 11794-8700 USA; 4 Omaha Veterans Affairs Medical Center (VAMC) and Nebraska Arthritis Outcomes Research Center, Division of Rheumatology, Department of Internal Medicine, College of Medicine, University of Nebraska Medical Center, 983025 Nebraska Medical Center, Omaha, NE 68198-3025 USA; 5 Department of Surgical Specialties, College of Dentistry, University of Nebraska Medical Center, 40th & Holdrege, Lincoln, NE 68583 USA

**Keywords:** Rheumatoid arthritis, Periodontitis, Alveolar bone loss, Case–control studies, Subantimicrobial dose doxycycline

## Abstract

In this review, we critically evaluate the case–control studies examining the relationship between rheumatoid arthritis (RA) and periodontitis, two common chronic inflammatory diseases with a similar host-mediated pathogenesis. We review the “two-hit” periodontitis model that our group previously proposed, in which we elucidate how a systemic disease such as RA can potentially exacerbate or initiate periodontitis. Furthermore, we discuss adjunctive host modulation therapy, originally developed for periodontitis (i.e., subantimicrobial-dose doxycycline alone or in combination with an anti-inflammatory agent), to simultaneously mitigate RA and periodontitis. Finally, we review studies describing periodontal treatment effects on both RA disease activity measures and systemic inflammation. Current evidence suggests that an association exists between periodontitis and RA. Well-designed multicenter longitudinal clinical trials and studies with sufficient sample sizes are needed to ascertain the temporal relationship between these two diseases and whether periodontal treatment can reduce the severity of RA or prevent its onset.

## Introduction

Periodontitis and rheumatoid arthritis (RA) are two common chronic inflammatory diseases sharing a similar host-mediated pathogenesis [1]. Periodontitis is characterized by soft and hard tissue destruction around teeth, ultimately leading to tooth loss [2], while RA is characterized by destruction of cartilage and bone in the joints, mediated by similar bone-resorptive cytokines and proteinases [1, 3]. Both diseases lead to significant morbidity, with periodontitis ultimately leading to tooth loss and loss of masticatory function, and RA leading to loss of joint function and loss of mobility.

Over the past 15 years, a number of case–control studies have suggested an association between the two diseases [4, 5••, 6–8, 9••, 10, 11]. Our group recently published a large case–control study examining the relationship between periodontitis and RA [5••]. By including a rigorously selected control group and addressing factors that may confound the relationship between RA and periodontitis, we endeavored to mitigate concerns regarding other published case–control studies. In the current review, we critically evaluate the studies examining the association between periodontitis and RA. Furthermore, we update our previously published “two-hit” periodontitis model, in which we hypothesize how a systemic disease such as RA can potentially exacerbate or even initiate periodontitis. We also discuss evidence supporting the potential use of subantimicrobial-dose doxycycline (SDD), a pharmacological therapy that is approved by the United States Food and Drug Administration (FDA) as well as regulatory agencies in Canada and the European Union, to treat periodontitis as an adjunct to mechanical debridement (scaling and root planing; SRP) and mitigate both diseases. We examine recently published periodontal intervention studies and their effect on RA disease activity measures, and we critically review their design. Finally, we discuss important questions that should be answered in future clinical trials, as further studies are needed to better understand the relationship between these two diseases.

## Clinical Studies Examining the Relationship Between RA and Periodontitis

In November 2012, a joint workshop was held by the European Federation of Periodontology and the American Academy of Periodontology on periodontitis and its relation to systemic diseases, including RA. At that time, virtually all of the studies on the association between RA and periodontitis that had been published had had fewer than 100 RA patients. Of those few studies with more than 100 RA patients, de Pablo et al. [6], had examined NHANES (National Health and Nutrition Examination Survey) III cross-sectional data, and had reported that the 103 RA patients in the sample were more likely than non-RA individuals (*n* = 4,358) to have missing teeth (mean of 20 vs. 16 missing teeth, *p* < 0.001), to have periodontitis (odds ratio [OR] 1.82, 95 % confidence interval [CI] = 1.04–3.20) and to be edentulous (OR = 2.27, 95 % CI = 1.56–3.31). Periodontitis was defined as the presence of at least one site with both attachment loss and probing depth ≥ 4 mm. This study was criticized in the joint workshop [12] because of the wide confidence intervals after adjusting for age, sex, race/ethnicity, and smoking, suggesting an imprecise population estimate.

Kaur et al. [13] published a systematic review, and included 16 case–control and 3 experimental studies that met their inclusion criteria. Based on ten of these studies that measured clinical attachment loss [7, 8, 14–21], the overall weighted mean difference (WMD) between clinical attachment loss in RA patients versus non-RA patients was 1.17 (95 % CI = 0.43–1.90), suggesting that attachment loss is greater in RA patients than in non-RA patients. However, only two of these studies [15, 18] had 100 or more RA participants. Kaur et al. [13] also reported that increased tooth loss was more common in individuals with RA than in those without RA, with a WMD of 2.38 (95 % CI =1.48–3.29), based on seven studies that met their criteria [7, 8, 15–17, 22, 23]. Only two of these studies had 100 or more RA patients [15, 22].

In a cross-sectional study not included in the Kaur et al. systematic review [13], de Smit et al. [11] reported a higher prevalence of severe periodontitis in RA patients (*n* = 95) compared to population-based, presumably healthy non-RA controls (*n* = 420) (27 % vs. 12 %; *p* <0.001). Severe periodontitis was defined based on the Dutch Periodontal Screening Index score. RA patients with severe periodontitis also had significantly higher DAS28 scores (28-joint disease activity score, a composite measure of RA disease activity) than RA patients without periodontitis or with moderate periodontitis, with no difference seen in IgM rheumatoid factor (RF) or anti-citrullinated protein antibody (ACPA) reactivity.

Shortcomings of these earlier studies included small sample size, lack of uniformity in defining periodontitis, and the preferential selection of healthy control groups, the latter serving as a potential source of bias. In 2014, our group [5••] published the largest case–control study to examine the association between periodontitis and RA that included a full-mouth periodontal examination to determine periodontitis status. In our study, 287 RA cases and 330 osteoarthritis (OA) controls were enrolled from four Veterans Administration Medical Centers and one academic coordinating medical center. Rather than enrolling healthy or non-arthritic controls, as in other studies, we enrolled OA patients as a non-inflammatory arthritis control group, with the expectation that these patients would be similar demographically to the RA cases.

Our study was rigorously designed, and addressed shortcomings in previous case–control studies. The comprehensive periodontal examination was conducted by a single examiner at each study center, and the examiners were calibrated prior to study initiation by a single periodontist. Each examiner was masked with respect to arthritis case status and results of any other clinical or laboratory evaluations. Probing depths and gingival recession were determined at six sites per tooth for all erupted teeth except for third molars. We defined periodontitis a priori according to the definition of Machtei et al. [24] as the presence of clinical attachment loss ≥ 6 mm on ≥ 2 teeth and one or more sites with probing depths ≥ 5 mm. This definition approximates the severe periodontitis definition based on the Centers for Disease Control/American Academy of Periodontology (CDC/AAP) classification [25], except that we included all six sites per tooth (rather than only the four interproximal sites) to determine the presence or absence of periodontitis. Although the CDC/AAP classification includes measurement of six sites per tooth, only the four interproximal sites are included in the periodontitis case definition, which may underestimate the prevalence of disease [26]. By defining periodontitis in our study as severe periodontitis, the likelihood of a false-positive diagnosis was reduced.

In our study, anti-cyclic citrullinated peptide (CCP)-positive RA patients were significantly more likely to have periodontitis than OA controls (37.1 % vs. 26.4 %, *p* = 0.006) [5••]. Based on a multivariable model that accounted for age, gender, smoking status, HLA-DRB1 shared epitope (major histocompatibility antigen associated with RA) positivity, race/ethnicity, body mass index, oral hygiene with supragingival plaque as a surrogate, self-reported diabetes mellitus, marital status, patient-reported oral dryness, and education level, the anti-CCP-positive RA patients remained significantly more likely to have periodontitis than controls (OR = 1.59, 95 % CI = 1.01–2.49; p = 0.043). Relative to RA patients without periodontitis, those with periodontitis also were significantly more likely to be positive for RF and had higher mean serum ACPA and RF concentrations. These associations were independent of the presence of visible supragingival plaque.

Regarding tooth loss in our study, there were 786 screening failures in the RA group and 936 screening failures in the OA group. Approximately one of every five (21.6 %) RA screening failures was due to total edentulism, while 17.9 % of the failures were due to the presence of fewer than nine posterior teeth, an exclusion criterion. In the OA group, only 8.0 % of the screening failures were due to total edentulism, while 8.2 % of the screening failures were due to the presence of too few teeth. These data suggest that tooth loss is more common in RA patients (relative to the OA control population), in agreement with Kaur et al. [13].

In addressing possible mechanisms, our study also examined the relationship between specific ACPAs and their association with alveolar bone loss [27••]. This focus was based, at least in part, on prior results from our group showing that ACPA concentrations were higher among RA patients with periodontitis [5••] and were positively correlated with the presence of antibodies to *Porphyromonas gingivalis* [28], a major etiologic risk factor associated with periodontitis, and the only prokaryote known to induce citrullination of RA-related neoantigens [29]. Following multivariate adjustment, increased alveolar bone loss was significantly associated with higher ACPA concentrations. In addition, ACPAs targeting citrullinated vimentin and histone were significantly increased in groups with moderate and high alveolar bone loss versus those with low levels of bone loss, regardless of smoking status. These data are important, as Harre et al. [30] recently reported that ACPA binding to citrullinated vimentin enhanced osteoclast differentiation into mature osteoclasts, suggesting a link between specific autoantibody production in RA and alveolar bone loss associated with periodontitis.

A gap in the literature that has been addressed in the past two years is the examination of an association between RA and periodontitis in patients with early treatment-naïve RA [9••, 10]. Similar to a study by Scher and colleagues involving treatment-naïve RA patients in the U. S., [10], Potikuri et al. [9••] examined this association in 91 non-smoking RA participants compared to 93 healthy controls (age- and sex-matched non-smoking patients) in India. Probing depths were measured, and periodontitis was deemed present if the mean pocket depth in a patient was ≥ 3 mm. The odds of having periodontitis were 4.28 times higher for patients with RA (95 % CI = 2.35–7.38; *p* < 0.001) compared to healthy controls (64.8 % vs. 28 %). Antibody titers to RF and ACPA were significantly higher in RA patients with periodontitis than in those without periodontitis. This case–control study is important for three reasons. First, the association between RA and periodontitis was observed in non-smoking patients, thereby eliminating smoking, a strong environmental risk factor for RA [31–33] and periodontitis [34, 35], as a potential confounding variable. Second, since biologics to treat RA can reduce gingival inflammation and may mitigate periodontitis [36], this confounder also was removed. Finally, in long-standing RA, a patient’s removal of supragingival plaque may be compromised by poor manual dexterity; by examining patients with early RA (average duration of RA was 25.1 months for ACPA-positive participants), the confounding relationship between heavy plaque accumulations, gingival inflammation, and periodontitis was potentially addressed, although plaque scores were not reported in this paper. As discussed earlier [5••], in the study in which we compared periodontitis prevalence in RA patients versus OA controls, poor manual dexterity was also likely eliminated as a confounding factor by accounting for plaque in the multivariable model. Thus, these studies [5••, 9••] further strengthen the evidence supporting an independent association between RA and periodontitis.

Similarly, Wolff et al. [37] examined the association between early RA and periodontitis in a small case–control study conducted in Germany. Only 22 early-RA patients and 22 controls were enrolled. The mean RA disease duration in this study was 5.9 months. Relative to the healthy controls (gender-, age- and smoking status-matched), early-RA patients had a greater number of missing teeth (5.7 vs. 1.9, *p* = 0.002), deeper periodontal pockets (2.9 mm vs. 2.4 mm, *p* < 0.0001), and a higher prevalence of bleeding on probing (18.6 % vs. 10.5 %, *p* = 0.001) with comparable oral hygiene between groups.

Dev et al. [38] conducted a cross-sectional study in which the prevalence of RA was compared in periodontitis versus non-periodontitis groups in India. The overall prevalence of RA was 4.4 % (37 of 852 participants) in the periodontitis group (defined based on the Community Periodontal Index), compared to 1 % prevalence in the general population. However, the prevalence of RA was not reported for the control group in this study; rather, the authors used the general population estimate of 1 %.

Among other studies published in 2014, Monsarrat et al. [39] assessed oral health in 74 RA patients, and Khantisopon et al. [40] did the same in 196 RA participants. However, neither study had a control group. Finally, in an Indonesian study, Susanto et al. [41] examined 75 RA patients and 75 controls, and found no difference in periodontitis prevalence between the two groups, although the RA participants had less healthy pocket epithelium versus controls based on the periodontal epithelial surface area score. However, the control group had an extremely high prevalence of moderate to severe periodontitis (69 %), which likely precluded detection of any differences relative to the RA group (prevalence = 71 %).

## Periodontal Host Modulation Therapy to Simultaneously Mitigate RA and Periodontitis

One obvious pathologic pathway common to both chronic periodontitis and RA is the excessive degradation of collagen-rich tissues: gingiva, periodontal ligament, and alveolar bone in periodontitis; and bone, cartilage, and other periarticular tissues in RA. The essential involvement of a unique category of host-derived tissue-destructive neutral proteinases in these two diseases, the collagenolytic matrix metalloproteinases (MMPs), including MMP-1 (collagenase-1), MMP-8 (collagenase-2), and MMP-13 (collagenase-3) (plus several other MMPs targeting this ubiquitous connective tissue molecule, collagen, such as MMP-2/gelatinase A and MMP-14/membrane-type MMP), has been addressed in several reviews [42–45]. In turn, this has led to novel pharmacological treatment strategies common to both RA and periodontitis, including the following:

(i) Collagenase or MMP inhibitors used as adjuncts to conventional antibacterial periodontal treatments (i.e., SRP to reduce the bacterial burden in the periodontal pocket); in this regard, SDD (a non-antibiotic tetracycline formulation) is an approved adjunctive treatment for periodontitis, and is the first-ever systemically administered MMP inhibitor drug approved by the FDA for any dental or medical disease [42, 44]; and
(ii) The same MMP inhibitor, SDD (20 mg doxycycline twice daily) for periodontitis, was found by O’Dell et al. [46] to be equally effective as the higher antibiotic dose of doxycycline (100 mg twice daily) in reducing the severity of early RA in a double-blind placebo-controlled clinical trial, except that the antibiotic-dose doxycycline, as expected, showed more adverse events (AEs) over the two-year protocol than the SDD treatment, which was similar to the placebo group with regard to the incidence of AE.

Of particular interest regarding the similar response of both periodontitis and RA patients to this anti-MMP strategy, both diseases also appear to benefit similarly from the combination of the tetracycline-based MMP inhibitor drug administered together with anti-inflammatory medications. For example, in the study by O’Dell et al. [46], the control RA patients were treated with standard-of-care methotrexate (a potent anti-inflammatory drug) combined with a placebo daily for two years. The two experimental groups of RA patients were also administered methotrexate, but in addition, received either antibiotic-dose doxycycline or non-antibiotic SDD as MMP inhibitory agents. The two MMP inhibitor formulations were substantially more effective in reducing global clinical scores (American College of Rheumatology 50 % improvement response, ACR50) of RA disease activity than methotrexate plus placebo. More specifically, a 50 % improvement in ACR response was observed in 41.6 % of patients receiving 100 mg doxycycline twice daily, in 38.9 % of individuals receiving 20 mg doxycycline twice daily, and in 12.5 % of participants receiving a placebo.

This apparent synergistic effect of the tetracycline-based MMP inhibitor combined with an anti-inflammatory agent was predicted by earlier studies on rats with experimental/adjuvant-induced RA [47, 48] as well as in humans with chronic periodontitis [49]. In the former, an adjuvant arthritic rat model demonstrated that a non-steroidal anti-inflammatory drug (NSAID) such as flurbiprofen only reduced signs of inflammation of the joints, and did not reduce the destruction of the collagen-rich tissues, bone and cartilage [47]. In contrast, arthritic rats treated only with a tetracycline-based MMP inhibitor (chemically modified tetracycline [CMT]-1, a non-antimicrobial tetracycline-based compound) showed decreased bone and cartilage destruction but no demonstrable anti-inflammatory effect. However, when CMT-1 was combined with flurbiprofen, a synergistic reduction in both joint inflammation and tissue destruction was observed [47]. Ramamurthy et al. [48] identified a mechanism for this synergistic response: the systemically administered NSAID appeared to increase the local/joint tissue uptake of the tetracycline compound by 116 % (delivered to the tissue from the circulation) compared to CMT-1 uptake by the arthritic tissues when this drug was administered alone.

A virtually identical synergistic response was seen in humans with severe periodontitis requiring surgical intervention. In a study by Lee et al. [49], patients who were administered a low dose (50 mg once daily) of flurbiprofen showed no reduction in MMP (collagenase and gelatinase) activity in gingival tissue extracts surgically excised for therapeutic purposes. In contrast, subjects treated with SDD showed significant reductions (as expected) in these host-derived collagenolytic proteinases. However, when the ineffective NSAID was combined with the effective SDD, a synergistic effect on MMP reduction was observed. As described above, this pattern of response to the combination therapy is consistent with the observations of O’Dell et al. [46] in patients with RA and in rats with experimental RA [47, 48], and provides additional evidence that both chronic periodontitis and RA are mediated by common pathological pathways that are suppressed in both diseases by similar MMP inhibitor therapeutic strategies.

**Fig. 1:**
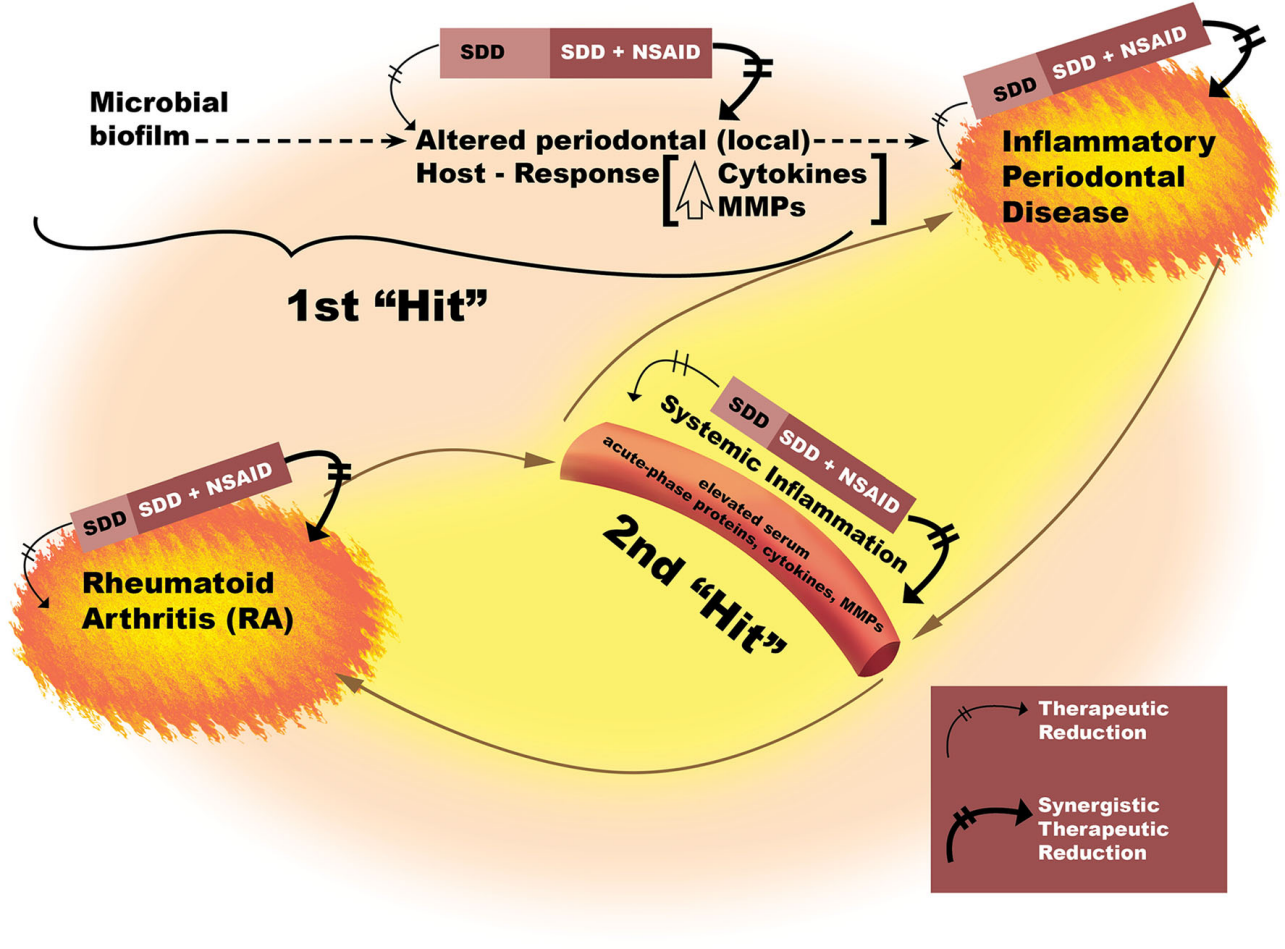
A modified “two-hit” model of induction of severe chronic periodontitis by local and systemic factors, its relationship to RA, and their mitigation by periodontal host modulation therapy. The first “Hit” involves the periodontopathic microbial biofilm and its metabolic products, such as lipopolysaccharide/endotoxin, inducing a local inflammatory response characterized by increased production in the periodontium of, particularly, bone-resorptive cytokines (IL-1, IL-6, TNF-α) and tissue-destructive proteinases (MMP-8, MMP-9, MMP-13, and neutrophil elastase). The second “Hit” involves a medical disease associated with destructive periodontitis (e.g., RA) which, like chronic periodontitis, can induce systemic inflammation characterized by elevated levels of acute-phase proteins such as C-reactive protein and other biomarkers/mediators in the circulation (e.g., IL-1, IL-6, TNF-α, MMP-8, and MMP-9). “Therapeutic reduction” represents the clinical/biological response to SDD host modulation therapy, adjunctive to SRP. “Synergistic therapeutic reduction” represents the response to the combination of SDD plus an anti-inflammatory drug (e.g., flurbiprofen), adjunctive to SRP. This figure is a modification of one published by us previously [50]

A “two-hit” model for the link between periodontitis and systemic diseases, including RA, was proposed by Golub et al. [50], and a modified version is presented in Fig. 1 (used with the permission of the *Journal of Dental Research*, SAGE Publications). It is currently recognized that both periodontitis and RA, when sufficiently severe or after sufficient duration of disease, are associated with systemic inflammation characterized by elevated circulating levels of acute-phase proteins—notably C-reactive protein (CRP) —and other biomarkers and mediators of inflammation (e.g., interleukin [IL]-6) and tissue destruction (e.g., MMP-9) [23, 50]. Mechanisms have been described that would enable each of these chronic inflammatory diseases, via an increase in systemic inflammation, to initiate or enhance the severity of the other. In this regard, several clinical trials in patients with chronic periodontitis and cardiovascular disease [51, 52], local (periodontal) and systemic bone loss (i.e., osteopenic postmenopausal women with chronic periodontitis [53]), or chronic periodontitis and type 2 diabetes [54] have shown that systemically administered SDD (as a pleiotropic MMP inhibitor pharmacologic), adjunctive to nonsurgical mechanical debridement periodontal therapy, can reduce the levels of these biomarkers/mediators of systemic inflammation (CRP, IL-6, and MMP-9) and collagenolysis, and benefit both the oral cavity and the medical condition. Given the efficacy of SDD as adjunctive therapy for periodontitis, one would expect that the two-year SDD regimen, which reduced the severity of early RA in O’Dell’s clinical trial [46], would have also reduced the severity of chronic periodontitis in these RA patients, although this was not part of their study. Clearly, further studies to confirm this hypothesis are warranted.

In addition to systemically administered SDD, which is non-antimicrobial but retains its anti-collagenolytic properties, the use of locally delivered antibacterial doxycycline as an adjunct to mechanical debridement during periodontal maintenance may be useful in treating not only periodontitis [55], but concomitant RA as well. We hypothesize that, since locally delivered sustained-release doxycycline has been shown to be effective against *Porphyromonas gingivalis* [56], the only known prokaryotic organism that possesses the peptidylarginine deiminase enzyme involved in the citrullination process [29, 57], reductions in subgingival *P. gingivalis* in response to local doxycycline therapy may result in less protein citrullination in the periodontium, and therefore less “spillover” of these proteins—or possibly locally produced ACPA—into the systemic circulation. The resolution of inflammation from the host modulatory effects of doxycycline may also indirectly alter the composition of the pathologic biofilm back to a healthy microflora, possibly by impacting the flow and composition of the gingival crevicular fluid environment in which the microbial shifts occur [58]. Since antibodies to citrullinated proteins are involved in the pathogenesis of RA [59], it is conceivable that this local and systemic host modulatory approach may be useful in treating RA and chronic periodontitis concurrently.

## Effects of Conventional Periodontal Treatment on RA Disease Activity and Systemic Inflammation

We identified four studies that were conducted prior to 2013 that had reported the effects of periodontal treatment on RA disease activity and systemic inflammation. These studies, as well as more recent ones, are summarized in Table 1. Limitations of the earlier studies included extremely small sample size and different periodontitis case definitions: Al-Katma et al. [60] diagnosed chronic periodontitis based on the International Workshop for a Classification of Periodontal Diseases and Conditions (Armitage criteria) [61], while the Ribeiro et al. [62] and Pinho et al. [63] studies used the Machtei et al. classification [24]; the periodontitis case definition was unspecified for Ortiz et al. [64].

**Table 1:** Periodontitis Intervention Studies in Rheumatoid Arthritis Patients

Study	Number of Patients and Periodontal Treatment	Design/Duration	Main Systemic Findings
Ribeiro et al. [62]	RA Control: n = 16, OHI + Supragingival Cleaning RA Experimental: n = 26, OHI + Supragingival Cleaning + SRP	− Randomization not specified − 3 months	↓ ESR in experimental group; No change in RF or HAQ
Al-Katma et al. [60]	RA Control: n = 12, no treatment RA Experimental: n = 17, OHI + SRP	− Randomized − No intent-to-treat analysis (i.e., dropouts not included in analysis) − 8 weeks	↓ ESR and DAS28 in experimental group compared with control group; No change in swollen or tender joint count, global well-being or morning stiffness
Pinho et al. [63]	RA Control: n = 15, no periodontal treatment RA Control: n = 15, full mouth extraction, no follow-up visit RA Experimental: n = 15, SRP 2 additional groups, n = 15 each, without RA	− Non-randomized − 6 months	No differences at 6 months between RA groups in ESR or other acute phase reactants or DAS28
Ortiz et al. [64]	RA Control: n = 20, no periodontal treatment, half on anti-TNF-α RA Experimental: n = 20, SRP + OHI + half on anti-TNF-α	− Randomized − 6 weeks	↓ DAS28, swollen and tender joints, global well-being, and TNF-α in group receiving periodontal treatment
Biyikoğlu et al. [66]	Non-RA patients: n = 15 RA patients: n = 15 Both groups received OHI + SRP	− Per protocol analysis, not intent-to-treat (only 10 RA patients and 12 non-RA patients completed the study) − 6 months	↓ DAS28 one month after SRP in RA patients, maintained at 3 and 6 months; ↓ CRP one month and three months after SRP; No change in RF, ESR
Erciyas et al. [67]	RA patients with moderate-to-high DAS28 scores, n = 30 RA patients with low DAS28 scores, n = 30 Both groups received OHI + SRP	− 3 months	↓ DAS28, ESR, CRP and TNF-α in both groups at 3 months
Okada et al. [68]	RA Control: n = 29, no periodontal treatment RA Experimental: n = 26, OHI + supragingival scaling	− Randomized − 8 weeks	Greater ↓ in DAS28-CRP and serum IgG to *P. gingivalis* and citrulline in the periodontal treatment group than in the control group; No change in swollen or tender joint count, global well-being, CRP, RF, or anti-CCP antibody

Key: *CCP* Cyclic citrullinated peptide, *CRP* C-reactive protein, *DAS28* Disease Activity Score (28 joints), *ESR* Erythrocyte sedimentation rate, *HAQ* Health Assessment Questionnaire, *OHI* Oral hygiene instructions, *RF* Rheumatoid factor, *SRP* Scaling and root planing, *TNF-α* Tumor necrosis factor-α

Since mid- 2013, three studies have examined the effects of periodontal treatment on DAS28, a commonly used composite measure of RA disease activity [65]. Biyikoğlu et al. [66] recruited RA patients with chronic periodontitis and systemically healthy non-RA chronic periodontitis patients (periodontitis identified using the Armitage criteria [61]). All patients received SRP (one visit per week for four weeks). DAS28 was significantly decreased one month after nonsurgical periodontal treatment in the RA group and remained stable for the remaining five months of the study. Study medication use was maintained throughout the study, implying that the periodontal treatment, not other RA-specific treatments, likely reduced DAS28 scores. Limitations of this study included the small samples size, failure to use intent-to-treat analysis, and the lack of a non-periodontally treated RA group with periodontitis.

Likewise, Erciyas et al. [67] reported on nonsurgical periodontal treatment effects on DAS28 in a three-month study. RA patients with moderate to high disease activity (DAS28 ≥ 3.2) and RA patients with low disease activity (DAS28 < 3.2) were enrolled; all patients had chronic periodontitis based on the Armitage criteria [61]. All patients received SRP (one visit per week for four weeks). Three months after nonsurgical periodontal treatment, DAS28 levels, serum erythrocyte sedimentation rate (ESR), CRP, and tumor necrosis factor (TNF)-α were statistically significantly lower in both groups. Of note, reductions in DAS28, ESR, and CRP in the moderate-to-high disease activity group were greater than those of the low-disease activity group. These authors reported that no medication changes were undertaken during the course of the study; thus, the reduction in DAS28 activity could not be explained by RA-specific treatments alone. Limitations of this study included the small sample size as well as the lack of a control group (an RA group without any periodontal treatment).

Okada et al. [68] recruited patients with RA and periodontitis (based on the Armitage classification [61]) and found that supragingival scaling at the baseline visit resulted in greater reductions in DAS28-CRP and serum IgG to *P. gingivalis* and citrulline than in the control group. Limitations of this study included a small sample size and inadequate treatment for periodontitis (supragingival scaling rather than SRP), thus likely underestimating the periodontal treatment effect.

All previously published periodontal treatment studies included mechanical therapy. No periodontal host modulation therapy (e.g., systemic SDD or local doxycycline application) was used. In addition, all earlier studies were small, diagnosed periodontitis based on different case definitions, had varying follow-up periods (six weeks [64] to six months [63, 66]), and had different or unspecified RA durations at baseline. To confirm the use of SRP alone or in conjunction with periodontal host modulation therapy as effective treatment modalities for reducing RA disease activity, sufficiently powered multicenter randomized clinical trials are needed, and these have not been done. The reader is referred to a recent systematic review/meta-analysis regarding the periodontal treatment effects on RA disease activity measures [69•].

Monsarrat et al. [70•] published a paper on the design of a clinical trial in order to examine the effect of periodontal treatment on the biological and clinical parameters of RA. Their proposed study will be an open-label randomized controlled trial including participants with both RA and periodontitis. The investigators plan to enroll a total of 40 individuals into two arms (intervention group including full-mouth SRP, followed by systemic antibiotics [amoxicillin or clindamycin, if allergic to penicillins, for seven days], oral hygiene instructions, and rinsing with 0.12 % chlorhexidine gluconate for 10 days after periodontal treatment). Patients will be followed for three months, and the same intervention will then be applied to the control group. The primary outcome of this study will be change in DAS28 score. Although the sample size will again be limited, this trial may provide important guidance for the design of larger clinical trials to determine whether periodontal therapy can improve clinical outcomes and quality of life in patients with active RA. The authors also plan to use multivariate analysis to account for center effects and any medication changes. A major drawback of this study is the use of amoxicillin or clindamycin adjunctive to SRP. This antimicrobial approach will likely have limited efficacy, as periodontal pathogens have been shown to be resistant to these antibiotics, and this approach will likely lead to increased antibiotic resistance in these patients [71].

## Conclusions and Future Directions

Numerous case–control studies have demonstrated an association between RA and periodontitis. Short-term clinical trials have shown that nonsurgical periodontal treatment can reduce RA disease activity and systemic inflammation, although these studies have had small sample sizes, and studies with larger sample sizes and longer-term follow-up are needed. Since SDD as an adjunct to SRP has been shown to effectively treat periodontitis [42], and to successfully improve the parameters of early RA [46], we propose inclusion of non-antimicrobial, systemically administered SDD alone or in combination with an anti-inflammatory agent and locally delivered doxycycline (for probing depths 5 mm and greater) in future clinical trials examining the effect of periodontal therapy on RA disease activity. All of these pharmaceutical approaches are adjunctive to SRP. Customizing treatment of periodontitis in RA patients based on their systemic inflammatory status (i.e., personalized medicine approach) should also be considered [72]. We hypothesize that the impact of periodontal treatment on RA may be greater in patients with more systemic inflammation and when SRP is used in combination with adjunctive periodontal host modulation therapy.

Further longitudinal studies are also needed to address the temporal relationship between these two diseases. Questions to address via longitudinal studies and clinical trials include: Does a diagnosis of periodontitis precede the presence of serum ACPA and clinical presentation of RA? Is periodontitis merely another manifestation of RA (i.e., another “joint”)? Can treatment of periodontitis prevent the onset of clinical RA in patients at risk for RA or in patients who are already positive for ACPA? Can periodontal treatment meaningfully ameliorate the symptoms of RA and reduce RA disease activity? Lastly, mechanistic studies need to be conducted to better understand the connection between these two diseases.
